# Enhancing the Water Flux and Antifouling Properties of PES Membranes via the Construction of a Bimetallic Polyphenol Network

**DOI:** 10.3390/polym18111326

**Published:** 2026-05-27

**Authors:** Yubin Lin, Xiaoxue Xiao, Wenqiang Deng, Wei Mao, Cui Wei, Jinghong Zhou

**Affiliations:** 1 Light Industry and Food Engineering College, Guangxi University, Nanning 530004, China; linyubin@st.gxu.edu.cn (Y.L.); 2416391006@st.gxu.edu.cn (W.D.); maowei@st.gxu.edu.cn (W.M.); 2 Guangxi Key Laboratory of Clean Pulp & Papermaking and Pollution Control, Nanning 530004, China; xiaoxiaoxue@boe.com.cn

**Keywords:** polyethersulfone membranes, tannic acid modified Ag-MOFs, bimetallic polyphenol networks, antibacterial membranes

## Abstract

High-performance polyethersulfone (PES) ultrafiltration membranes integrating antibacterial activity and antifouling performance were fabricated via the in situ construction of bimetallic polyphenol networks (BMPNs) throughout the membrane architecture. Tannic acid (TA) functioned as a multifunctional molecular bridge, functionalizing silver metal–organic frameworks (Ag-MOFs) to yield hydrophilic T-Ag-MOFs and chelating Fe^3+^ ions from the coagulation bath to form a polyphenol network during phase inversion. T-Ag-MOF incorporation generated asymmetric morphologies featuring highly porous surfaces and sponge-like cross-sections, improving pure water permeability, mechanical integrity, and bovine serum albumin (BSA) rejection. TA-mediated functionalization increased hydrophilicity, imparted a negative surface charge, suppressed nonspecific protein adhesion, and enhanced flux recovery with low irreversible fouling. At an optimal loading of 0.4 wt%, the resultant T-Ag-MOF/Fe^3+^/PES composite membrane achieved a pure water permeability of 593.4 L m^−2^ h^−1^ bar^−1^—1.77-fold higher than that of the pristine PES control—while sustaining a BSA rejection of 96.5%. Notably, interfacial compatibility between the T-Ag-MOFs and PES matrix was enhanced, facilitating strong, covalent-like filler–matrix adhesion. Moreover, the composite membrane delivered synergistic multifunctionality, including exceptional long-term aqueous stability, precisely tuned Ag^+^ release kinetics, and potent antibacterial activity, as evidenced by negligible uncontrolled ion leaching and a lack of structural degradation under prolonged hydration.

## 1. Introduction

Membrane separation technology is among the most widely adopted approaches for water purification, valued for its high efficiency in bioseparation and protein recovery, oil–water emulsion separation, and its broad range of industrial applications [1,2]. Polyethersulfone (PES) is a benchmark polymeric material for municipal and industrial water treatment, characterized by its good mechanical strength, thermal and chemical stability, separation performance, and compatibility with low-pressure operation [3]. However, similar to many other conventional filtration membranes, commercial PES membranes are constrained by the fundamental permeability–selectivity trade-off, wherein a higher selectivity (i.e., retention) typically comes at the expense of reduced permeability, and vice versa [4]. Additionally, membrane fouling—particularly biofouling—remains a critical operational challenge, as the intrinsic hydrophobicity of PES promotes microbial adhesion and subsequent biofilm formation [5]. Over time, biofouling constricts the effective pore size, diminishes permeability, compromises separation performance, and ultimately shortens membrane service life [6,7]. As a result, concurrently enhancing membrane hydrophilicity and permeability significantly improves the overall antifouling performance [8]. For instance, surface and bulk modification strategies—including blending [9], coating [10], and grafting [11]—enhance hydrophilicity, suppress fouling, and impart bactericidal functionality. Among these, blending is the most widely used method, wherein inorganic hydrophilic additives—such as graphene oxide [12], carbon nanotubes [13], and metallic nanomaterials (e.g., Ag and Cu) [14,15]—are incorporated into the PES matrix. This synergistic integration leverages the inherent mechanical toughness of the polymer host and the superior hydrophilicity of the nanofiller, significantly improving both hydrophilicity and antifouling performance [16]. Nevertheless, these inorganic additives present several inherent limitations that pose significant challenges to both membrane fabrication and long-term performance. First, poor interfacial compatibility between inorganic additives and organic membrane matrices can lead to nanoparticle agglomeration and non-uniform dispersion, thereby diminishing the effectiveness of hydrophilic modification. Second, this incompatibility often results in nanoparticle leaching during extended filtration operation, which compromises membrane structural integrity, antifouling durability, and antibacterial efficacy [17,18,19]. Consequently, there is an urgent requirement to develop nanomaterials that exhibit both excellent compatibility with the polymeric matrix and stable, homogeneous dispersion [20].

Metal–organic frameworks (MOFs) are crystalline, porous materials composed of metal ions or clusters coordinated with organic ligands [21]. Owing to their high surface area, tunable porosity, and structural versatility, MOFs have been widely applied in adsorption [22], energy storage (e.g., supercapacitors [23]), catalysis [24], environmental remediation [25], and antibiofouling membrane modification. For instance, Alirera et al. [26,27] demonstrated that an Ag-MOF mitigates biofouling in forward-osmosis thin-film composite (TFC) membranes. Specifically, the incorporation of both graphene oxide (GO) and Ag-MOFs into the TFC selective layer produced a thin-film nanocomposite membrane with strong synergistic antibiofouling and antifouling properties [28]. However, the intrinsic hydrophobicity of many pristine MOFs not only hinders uniform dispersion within the polymer matrix but also promotes leaching during operation, thereby compromising the stability and continuity of the hydrophilic surface layer [17]. Therefore, rational functionalization and hydrophilic modification of MOFs are essential to enhance polymer–filler compatibility, stabilize their incorporation, and fully exploit their unique structural and functional advantages.

Tannic acid (TA) is a naturally occurring, branched, star-shaped polyphenol composed of five galloyl groups bound to a central glucose core via ester bonds [29]. These abundant pyrogallol units enable robust, multivalent interfacial binding to diverse substrates via concurrent hydrogen bonding, electrostatic attractions, π–π stacking, metal coordination, cation–π interactions, and dynamic covalent chemistry [30,31]. Critically, the high density of phenolic hydroxyl groups in TA facilitates rapid, aqueous-phase formation of stable metal–polyphenol networks (MPNs) with multivalent metal ions (e.g., Fe^3+^, Cu^2+^, and Ag^+^) [30]. For example, Fan et al. [32] and Kim et al. [33] fabricated TA–Fe^3+^-coated PES composite membranes via a rapid, one-step aqueous assembly process. These modified membranes exhibited enhanced structural stability, oxidative resistance, antifouling performance, and efficient heavy metal removal capacity. Additionally, Guo et al. [34] functionalized commercial nanofiltration (NF) membranes with TA–Fe^3+^ complexes, resulting in significantly enhanced rejection of NaCl and trace organic contaminants. While recent studies [35] have shown that TA can etch MOFs and promote in situ MPN formation—thereby improving the dispersibility, hydrophilicity, and stability of MOFs in polymeric systems—the deliberate construction of bimetallic polyphenol networks (BMPNs, e.g., Fe^3+^/Ag^+^–TA) within the bulk phase of PES membranes remains unexplored. Moreover, the integrated effects of such embedded BMPN architectures on membrane hydrophilicity, water permeability, antifouling performance, and controlled Ag^+^ release have yet to be systematically investigated.

**Figure 1:**
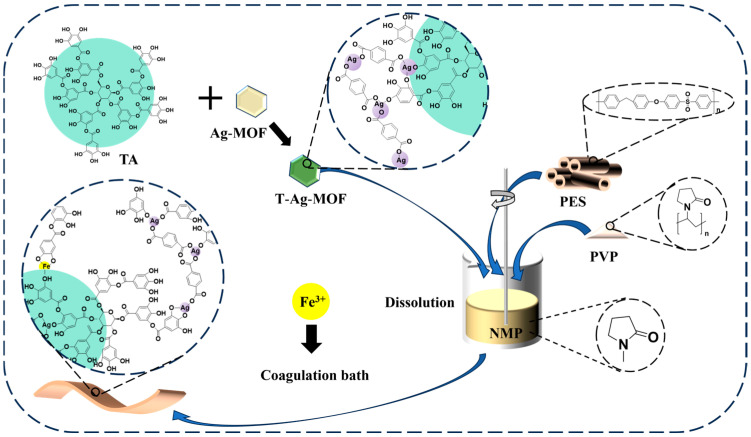
Schematic representation outlining preparation of the T-Ag-MOF/Fe^3+^/PES composite membrane.

This study presents a novel approach for fabricating high-performance water purification membranes through the construction of BMPNs within a PES matrix, as outlined in Figure 1. TA serves a dual functional role: its abundant phenolic hydroxyl groups act both as coordination sites for metal ions and as hydrophilic moieties that enhance the surface polarity of Ag-MOFs, yielding TA-functionalized Ag-MOFs (T-Ag-MOFs). Specifically, TA selectively etches pristine Ag-MOFs under mild acidic conditions, generating hydrophilic T-Ag-MOFs nanofillers. Subsequently, during membrane phase inversion, Fe^3+^ originating from the coagulation bath chelates with surface-exposed TA moieties on the T-Ag-MOFs, forming a secondary Fe^3+^–polyphenol coordination shell, and establishing a bimetallic architecture within the PES. Specifically, the BMPN structure is formed through three synergistic processes: (i) TA-mediated etching and functionalization of the T-Ag-MOFs, (ii) Fe^3+^ chelation with surface TA during phase inversion, and (iii) immobilization of T-Ag-MOFs via interpenetrating BMPN crosslinks throughout the PES matrix. This design overcomes two limitations in mixed-matrix membrane development, namely, poor interfacial compatibility between inorganic nanofillers and polymeric matrices, and filler leaching and hydrophilicity loss under prolonged aqueous operation. Subsequently, the influence of the T-Ag-MOF loading on membrane morphology, pure water permeability, and antibacterial efficacy are investigated. Additionally, the BMPN stability is rigorously evaluated by quantifying Ag^+^ release from the T-Ag-MOF/PES membranes and comparing it against control membranes containing unmodified Ag-MOFs. Finally, the hydrophilicity, antifouling performance, and antimicrobial activity of the BMPN-integrated PES membrane are evaluated and compared with those of conventional MPN-coated and pristine Ag-MOFs-doped membranes.

## 2. Materials and Methods

### 2.1. Materials

All chemicals were of analytical grade and were used as received unless otherwise stated. PES (Ultrason E6020P, Mw = 58,000 g mol^−1^) was supplied by BSAF (Nienburg, Germany). Bovine serum albumin (BSA, Mw = 66,000 g mol^−1^), TA (ACS reagent), and *p*-phthalic acid (PTA) were purchased from Aladdin Reagent Co., Ltd. (Shanghai, China). FeCl_3_·6H_2_O, AgNO_3_, urea, *N*,*N*-dimethylformamide (DMF), polyvinylpyrrolidone (PVP; molecular mass = 10,000 g mol^−1^), *N*-methyl-2-pyrrolidone (NMP, 99%), and 4-dimethylaminobenzaldehyde (PDAB) were obtained from Macklin Biochemical Co., Ltd. (Shanghai, China). *Escherichia coli* (MTCC-1687) and *Staphylococcus aureus* (MTCC-3160) were procured from Beijing Microbiological Culture Collection Center Co. (Beijing, China).

### 2.2. Preparation of the Ag-MOFs and T-Ag-MOFs

The Ag-MOFs were synthesized according to a modified solvothermal microwave-assisted method adapted from Tan et al. [19]. Specifically, AgNO_3_ (0.340 g) and PTA (0.332 g) were dissolved in DMF (40 mL) under magnetic stirring. The resulting mixture was transferred to a Teflon-lined stainless-steel autoclave and subjected to microwave irradiation (MW-1000, Nanjing Xian’ou Instrument Manufacturing Co., Ltd., Nanjing, China) at 140 °C for 90 min, and the power was set to 800 W. After this time, the autoclave was naturally cooled to 25 °C. The precipitate was collected by centrifugation, washed sequentially five times with anhydrous ethanol and deionized water to remove residual reagents, and dried under vacuum at 80 °C for 12 h to yield the desired Ag-MOFs.

The T-Ag-MOFs were prepared via post-synthetic modification of the Ag-MOFs according to previously reported protocols [36,37]. Specifically, the as-synthesized Ag-MOFs (0.1 g) were dispersed in a TA solution (100 mL, 1.0 g L^−1^) and stirred vigorously for 30 min at 25 °C. The suspension was then subjected to centrifugation (12,000 rpm, 10 min) and the resulting brown precipitate was collected, washed once with deionized water, and dried under vacuum at 80 °C for 12 h to yield the T-Ag-MOFs.

### 2.3. Membrane Preparation

The BMPN membranes were fabricated via a nonsolvent-induced phase separation (NIPS) technique [38]. The precise compositions of the casting solutions and coagulation baths are summarized in Table 1. According to the specified composition, PVP and the MOF were dispersed in NMP and subjected to ultrasonication for 10 min. Subsequently, PES and PVP were added to the dispersion, followed by vigorous stirring at 60 °C for 12 h. The resulting casting solution containing the homogeneously dispersed MOF was then defoamed for 24 h before scraping evenly onto a clean glass plate using a doctor blade with a gap of 200 μm. Immediately thereafter, the glass plate was immersed in a coagulation bath containing Fe^3+^ at pH 1.64. After phase conversion was complete, the film on the glass plate detaches and separates, and the nascent membranes were transferred to a deionized water bath for 72 h, with the deionized water changed twice daily. The resulting composite membranes were denoted as MP_5_, MTA_0.1_, MTA_0.2_, MTA_0.3_, MTA_0.4_, MTA_0.4_*, and MA_0.4_, as detailed in Table 1.

### 2.4. Characterization of the MOFs

X-ray diffractometry (XRD, Bruker D8 diffractometer, Bruker, Berlin, Germany) was employed to characterize the crystalline phases and structural properties of the synthesized Ag-MOFs and T-Ag-MOFs specimens. Zeta (ζ) potential measurements (Zetasizer Nano ZS90, Malvern, Worcestershire, UK) were performed for 0.02 wt% aqueous dispersions of the Ag-MOF and T-Ag-MOF samples. Static water contact angles (WCAs) were measured on vacuum-dried, freestanding Ag-MOF and TA-Ag-MOF membranes (thickness ≈ 80–100 μm), using a contact angle goniometer (Phoenix Mini, SEO, Seoul, Republic of Korea). A droplet of deionized water (5 μL) was gently placed on the membrane surface, and the contact angle was determined automatically from CCD-captured images using SurfaceWare software (v. 4.2.1), based on ≥4 independent measurements at randomly selected locations.

### 2.5. Membrane Characterization

The morphologies and microstructures of the Ag-MOFs, T-Ag-MOFs, and fabricated membranes were characterized using field-emission scanning electron microscopy (FE-SEM, Gemini 300, ZEISS, Oberkochen, Germany) and transmission electron microscopy (TEM, Talos F200S, Thermo Scientific, Waltham, MA, USA). The WCAs of the Ag-MOFs, T-Ag-MOFs, and membrane surfaces were measured using a Krüss DSA100 contact angle analyzer (KRÜSS Scientific, Hamburg, Germany).

### 2.6. Filtration Tests

Pure water flux (PWF) and BSA rejection were evaluated using an ultrafiltration cell (Model 8050, Millipore Corporation, Burlington, MA, USA) with an effective membrane area of 12.56 cm^2^. Prior to measurement, each membrane was compacted at 10^5^ Pa for 30 min to minimize irreversible compaction effects. The PWF was determined by collecting and measuring the permeate volume after 30 min of steady-state filtration at 1 bar. For BSA rejection testing, the feed solution was replaced with a 1.0 g L^−1^ BSA solution prepared in deionized water. To mitigate concentration polarization, the feed solution was stirred continuously at 300 rpm. The BSA concentrations in the feed and permeate streams were quantified using ultraviolet–visible (UV–vis) spectrophotometry at 280 nm.

The PWF (J) was calculated according to the following equation:

$$J\;=\;\frac{V}{t\;\times \;S\;\times \;∆P}$$

where J denotes the PWF (L m^−2^ h^−1^ bar^−1^), V is the permeate volume (L), S is the effective membrane area (m^2^), t is the filtration duration (h), and ΔP is the applied transmembrane pressure (bar).

The membrane fouling behavior during BSA filtration was quantified in terms of the reversible fouling ratio (R_r_), irreversible fouling ratio (R_ir_), and total fouling ratio (R_t_), which were calculated as follows:

$$\mathrm{FRR}\;(\%)\;=\frac{J_{2}}{J_{0}}\;\times \;100$$

$$R_{t}\;(\%)=\frac{{(J_{0}-J}_{1})}{J_{0}}\times 100$$

$$R_{r}\;(\%)=\frac{{(J_{2}-J}_{1})}{J_{0}}\times 100$$

$$R_{\mathrm{ir}}\;(\%)=\frac{{(J_{0}-J}_{3})}{J_{0}}\times 100$$

where J_0_ denotes the initial PWF (L m^−2^ h^−1^ bar^−1^), J_1_ represents the steady-state flux (L m^−2^ h^−1^ bar^−1^), and J_2_ is the recovered PWF after a 3 h backwash (L m^−2^ h^−1^ bar^−1^).

The equation used to determine the membrane retention of BSA (R, %) is shown below:

$$R\;(\%)\;=\;\frac{C_{f}-C_{p}}{C_{f}}\;\times \;100$$

where C_p_ and C_f_ are the BSA concentrations (g L^−1^) of the filtrate and feed solution, respectively.

### 2.7. Antibacterial Assays

The antibacterial activity of each MOF against *E. coli* and *S. aureus* was evaluated using two complementary methods, namely, the agar diffusion (inhibition zone) assay and planktonic growth inhibition assay. Bacterial cultures were grown in Luria–Bertani (LB) broth at 37 °C for 24 h, then diluted with sterile phosphate-buffered saline (PBS) to yield a final concentration of ~10^4^ CFU mL^−1^ (inhibition zone assays) or ~10^6^ CFU mL^−1^ (growth curve assays).

#### 2.7.1. Inhibition Zone Assays

An aliquot (100 μL) of the standardized bacterial suspension (1.0 × 10^4^ CFU mL^−1^) was uniformly spread onto the surface of a pre-poured, fully solidified LB agar plate (1.5% *w*/*v*). Sterile membrane discs (6 mm diameter), loaded with the test samples, were placed aseptically onto the inoculated agar surface and incubated at 37 °C for 24 h under aerobic conditions. The antimicrobial efficacy was quantified as the diameter (mm) of the clear inhibition zone surrounding each disc, measured using ImageJ software (v1.53t, NIH) [39]. Measurements were performed in triplicate; the results are reported as the mean ± standard deviation (SD).

#### 2.7.2. Planktonic Growth Inhibition Assay

Sterile LB broth (100 mL) was inoculated with the standardized bacterial suspension (100 μL, 1.0 × 10^6^ CFU mL^−1^) and supplemented with membrane samples at a final concentration of 0.5 mg mL^−1^. Control cultures were also prepared, containing identical volumes of bacteria and LB broth but no test material. The optical density at 600 nm (OD_600_) was measured at 0, 2, 4, 6, 12, and 24 h using UV–vis spectroscopy (UV1901PC, Shanghai, China), with sterile LB broth serving as the blank reference.

### 2.8. Silver Ion Leaching Assay

Each modified membrane sample (0.05 g, MA_0.4_ and MTA_0.4_) was cut into 1.5 cm × 1.5 cm pieces and immersed in ultrapure deionized water (200 mL, resistivity ≥ 18.2 MΩ·cm). The mixture was shaken at 150 rpm and 25 °C for 24 h. At times of 0, 2, 6, 10, 14, and 24 h, samples (5 mL) of the leachate were removed, filtered through 0.22 μm nylon filters, and analyzed for Ag^+^ using flame atomic absorption spectroscopy. All measurements were performed in triplicate; the results are reported as the mean ± SD.

## 3. Results and Discussion

### 3.1. Characterization of the T-Ag-MOF Particles

**Figure 2:**
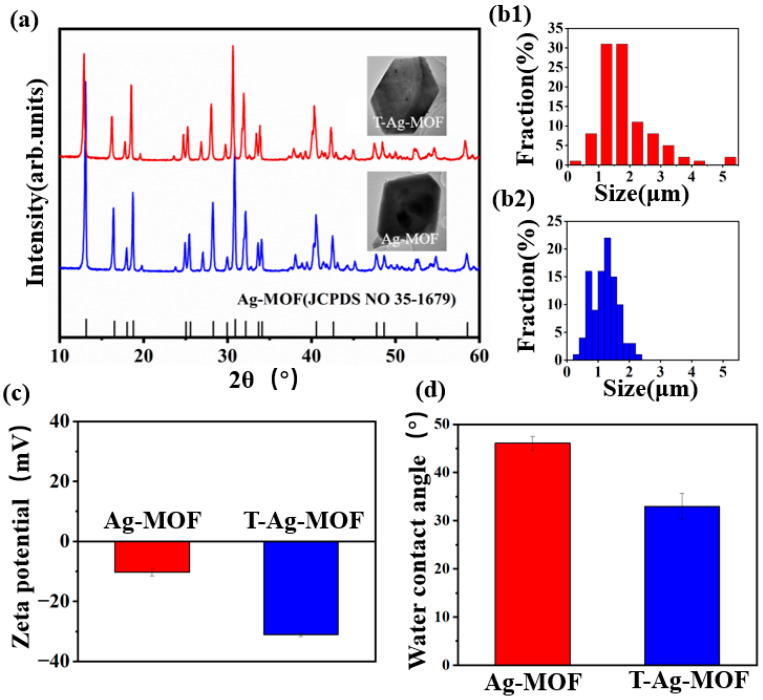
(**a**) XRD patterns and TEM images of the Ag-MOF and T-Ag-MOF specimens. Particle size distribution curves for (**b1**) Ag-MOF and (**b2**) T-Ag-MOF. (**c**) ζ-potentials and (**d**) WCAs of Ag-MOF and T-Ag-MOF.

The morphologies of the pristine Ag-MOFs and TA-functionalized Ag-MOFs (T-Ag-MOFs) were characterized by TEM. As shown in Figure 2a, the as-synthesized Ag-MOFs exhibit well-defined, hexahedron-like crystals with smooth surfaces and sharp edges. Additionally, their particle size distribution spans ~0.25–4.25 μm (Figure 2(b1,b2)). Following TA-mediated etching, the T-Ag-MOFs retained the characteristic hexahedral framework morphology—indicating preservation of the overall structural integrity—while exhibiting a reduced particle size (0.25–2.50 μm; Figure 2(b2)) and a markedly narrower size distribution, reflecting improved particle uniformity. Particle size is a critical structural determinant that governs both the morphological development of composite membranes during phase inversion and their resulting filtration performance, including water permeability, solute rejection, and fouling resistance [40,41].

The crystalline phase purity and structural retention of the prepared MOFs were further assessed by XRD (Figure 2a). The XRD pattern of the pristine Ag-MOFs matched well with the reference pattern JCPDS No. 36-1679 [42], confirming phase consistency with previously reported Ag-MOF structures. Distinct diffraction peaks were observed at 2θ = 13.2°, 16.5°, 18.1°, 18.8°, 25.1°, 25.6°, 27.1°, 28.3°, 29.9°, 30.9°, 32.2°, 33.7°, 34.1°, 40.6°, and 42.6°, which can be indexed to the (100), (110), (011), (111), (021), (111), (211), (210), (112), (121), (012), (031), (131), (100), and (302) crystallographic planes, respectively [43,44]. Following TA treatment, the XRD pattern of T-Ag-MOF exhibited a moderate decrease in peak intensities across all reflections, with no observable peak broadening or emergence of new diffraction features. This intensity attenuation was attributed to partial surface amorphization and localized lattice disorder, which are induced by the weakly acidic and polyphenolic chelating action of TA, without compromising bulk crystallinity.

Additionally, the surface physicochemical properties were evaluated via ζ-potential and WCA measurements (Figure 2c). As shown, TA functionalization induces a substantial negative shift in the ζ-potential—from −10.3 mV for the pristine Ag-MOF to −31.1 mV for T-Ag-MOF—alongside a marked reduction in the WCA from 46.1° to 32.9° (Figure 2d). These concurrent changes provide robust evidence for the successful surface coating of TA on the Ag-MOF structure. Furthermore, the enhanced hydrophilicity and increased surface charge density of T-Ag-MOF improve interfacial compatibility with the PES matrix and mitigate electrostatic repulsion during membrane fabrication, thereby promoting more uniform particle dispersion and stronger polymer–filler interactions.

### 3.2. Morphologies of the Modified Membranes

**Figure 3:**
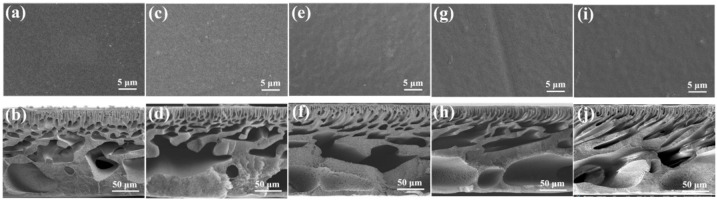
SEM images of the top-surface and cross-sectional morphology of T-Ag-MOF-modified membranes prepared at various loadings: (**a**,**b**) MP_5_ (pristine PES), (**c**,**d**) MTA_0.1_, (**e**,**f**) MTA_0.2_, (**g**,**h**) MTA_0.3_, and (**i**,**j**) MTA_0.4_.

Figure 3 presents SEM images of the surface morphologies and cross-sectional microstructures of the composite membranes fabricated with varying T-Ag-MOFs loadings. The pristine PES membrane (blank control, MP_5_) exhibits a dense, featureless surface and a cross-section comprising short, vertically aligned finger-like pores that terminate in a compact, sponge-like sublayer. Additionally, numerous closed pores are observed within these finger-like channels. In contrast, all T-Ag-MOF-modified membranes retain the canonical asymmetric architecture typical of nonsolvent-induced phase inversion membranes. Upon increasing the T-Ag-MOF loading from 0.1 to 0.4 wt%, the finger-like pores progressively elongate toward the lower surface of the membrane, accompanied by a marked increase in macrovoid volume and the emergence of well-defined spherical cavities. This morphological evolution is mechanistically linked to the enhanced hydrophilicity of the T-Ag-MOF—conferred by the abundant surface-bound phenolic hydroxyl groups introduced via TA functionalization—which reduces the thermodynamic affinity between PES and the casting solvent. Consequently, the critical water concentration required to trigger liquid–liquid de-mixing decreases, accelerating solvent–water exchange and promoting rapid water influx into the nascent polymer-rich phase [45,46]. The resulting T-Ag-MOF/Fe^3+^/PES composite membrane exhibits an enlarged, highly interconnected macrovoid network in the top selective layer and a transformed underlying sublayer featuring increased pore size, reduced packing density, and hierarchical porosity.

Moreover, TA serves not only as a hydrophilic modifier but also as a multifunctional interfacial mediator. Specifically, its catechol and galloyl moieties engage in synergistic coordination with Fe^3+^ ions in the coagulation bath and concurrent hydrogen bonding with both the chains of the PES backbone and T-Ag-MOF structure. This dual-affinity interaction drives the in situ formation of a robust, TA-enriched BMPN anchored at the membrane surface and uniformly distributed across the pore walls, thereby simultaneously enhancing bulk porosity, surface hydrophilicity, and interfacial cohesion.

### 3.3. Membrane Performance

**Figure 4:**
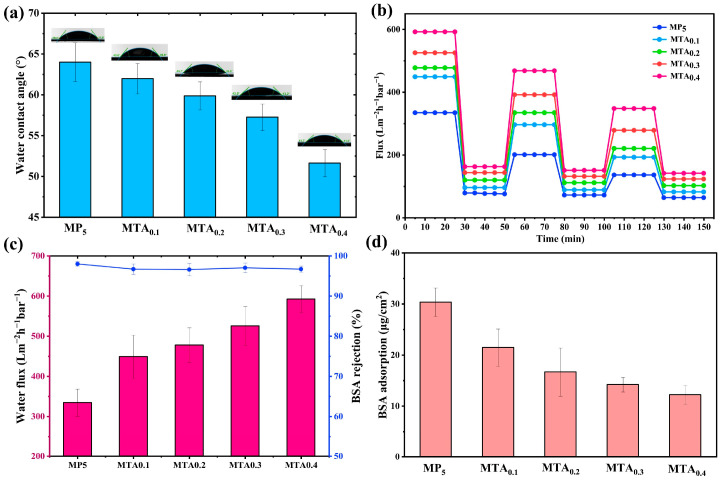
Properties of the modified membranes: (**a**) WCA, (**b**) time-dependent flux, (**c**) BSA rejection, and (**d**) BSA adsorption of the different membranes.

The antifouling performance was quantitatively assessed via the flux recovery ratio (FRR), with higher FRR values indicating enhanced resistance to irreversible fouling. As detailed in Table 2, all T-Ag-MOF/Fe^3+^-modified membranes exhibit substantially higher FRR values relative to the unmodified membrane (MP_5_). Moreover, increasing the T-Ag-MOFs loading leads to a gradual rise in both the total fouling ratio (R_t_) and reversible fouling ratio (R_r_), while the irreversible fouling ratio (R_ir_) consistently decreases, consistent with the observed reduction in WCA (Figure 4a). This behavior is primarily attributable to the improved surface hydrophilicity of the modified membranes, which plays a critical role in mitigating fouling. Specifically, the abundant hydrophilic functional groups (e.g., catechol and phenolic hydroxyl groups) introduced by TA facilitate strong hydrogen-bonding interactions with water molecules, forming a robust hydration layer that impedes the direct adsorption of BSA. Good hydrophilicity therefore helps improve the antifouling performance of the membrane and can significantly extend its service life. Figure 4b presents the time-dependent permeation flux profiles for pure water and the BSA solution over three consecutive filtration cycle. As shown, the PWF of all T-Ag-MOF-modified membranes is markedly enhanced relative to that of the pristine MP_5_ membrane. Notably, PWF increases progressively with T-Ag-MOF content, with the MTA_0.4_ membrane achieving the highest permeability of 593.36 L m^−2^ h^−1^ bar^−1^, which represents a 1.77-fold enhancement over MP_5_ (335.2 L m^−2^ h^−1^ bar^−1^). During each BSA filtration cycle (1.0 g L^−1^), all membranes exhibit an initial flux decline attributable to concentration polarization and reversible adsorption. Critically, however, the MP_5_ membrane displays progressive irreversible fouling accumulation across cycles, reaching a final FRR of 40.62% after the third cycle. In contrast, the MTA_0.4_ membrane maintains exceptional cycling stability—achieving an FRR of 58.64% even after three repeated fouling–cleaning cycles. Additionally, the PWF decay profile of MP_5_ exhibits a significantly steeper decline than those of the modified membranes, further supporting the superior antifouling performance conferred by T-Ag-MOF incorporation. These results confirm that the antifouling performance conferred by T-Ag-MOF incorporation is robust, reproducible, and sustained under operational conditions involving multiple fouling–regeneration events, and is not merely an artifact of a single-cycle measurement. This dual enhancement in permeability and fouling resistance arises from two synergistic mechanisms. Firstly, the homogeneous dispersion of T-Ag-MOF nanoparticles within the PES matrix promotes favorable phase inversion kinetics, yielding elongated finger-like macrovoids and suppressing the formation of irregular large voids (Figure 3). Secondly, the TA-mediated partial etching of Ag-MOFs not only enhances nanoparticle hydrophilicity and surface charge density (as evidenced by the increased ζ-potential) but also introduces additional hydroxyl-rich moieties onto the membrane surface, collectively augmenting interfacial hydration, water transport efficiency, and resistance to protein adhesion.

BSA rejection, which represents a well-established indicator of membrane selectivity and structural integrity, was subsequently evaluated for the pristine MP_5_ control membrane, which exhibited a high BSA rejection of 97.99%. Upon T-Ag-MOF incorporation, the BSA rejection of the T-Ag-MOF/Fe^3+^/PES composite membranes decreased slightly but remained consistently above 96.5%, demonstrating that nanoparticle integration preserves the intrinsic separation capability of the membrane without inducing structural defects or compromising integrity. Specifically, MTA_0.1_, MTA_0.2_, MTA_0.3_, and MTA_0.4_ achieved rejection capabilities of 96.70%, 96.59%, 97.03%, and 96.70%, respectively. This minimal variation is corroborated by top-surface SEM analysis (Figure 3), which revealed no discernible pores on any membrane surface, thereby confirming the intact selective layered morphology and sustaining robust size-exclusion performance. Cross-sectional SEM images further reveal distinct microstructural features: MP_5_ displays a relatively dense support layer with numerous closed finger-like pores, impeding both water permeation and BSA transport; in contrast, the T-Ag-MOF/Fe^3+^/PES membranes exhibit well-connected, open finger-like pores and a thinner, more porous sponge-like sublayer, ultimately enhancing hydraulic permeability while maintaining high solute retention. Static BSA adsorption assays (Figure 4d) reveal that the unmodified MP_5_ membrane adsorbs 30.34 μg cm^−2^ BSA. In contrast, T-Ag-MOF incorporation leads to a pronounced, dose-dependent suppression of protein adsorption, with the corresponding values decreasing progressively to 21.46, 16.67, 14.20, and 12.20 μg cm^−2^ at T-Ag-MOF loadings of 0.1, 0.2, 0.3, and 0.4 wt%, respectively, indicating improved antifouling potential.

**Figure 5:**
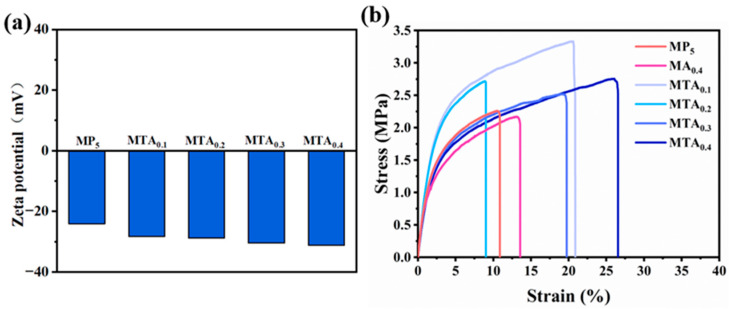
(**a**) Zeta potentials and (**b**) Stress–strain curves of the MP_5_ and TA/Fe^3+^/Ag-MOF-modified membranes (MTA_0.1–0.4_).

Critically, ζ potential measurements (Figure 5a) reveal that all T-Ag-MOF/Fe^3+^/PES membranes exhibit more negative surface charges (−28.3 to −31.2 mV) than the pristine MP_5_ membrane (−24.1 mV) across the pH range relevant to BSA filtration (pH 5–7). This finding is also supported by the contact angle measurements (Figure 4a). Given that BSA carries a net negative charge under these conditions (pI ≈ 4.7), the increased negative surface charge of the modified membranes strengthens electrostatic repulsion against approaching BSA molecules, thereby suppressing adsorption and mitigating irreversible fouling. This synergistic effect—combining enhanced hydration-layer-mediated steric hindrance with augmented electrostatic repulsion—therefore constitutes a dual-mechanism antifouling strategy [47].

Figure 5 presents the uniaxial tensile stress–strain curves of PES-based membranes with varying T-Ag-MOF loadings. The data reveal a nonmonotonic dependence of mechanical performance on filler content: tensile strength and elongation at break both increase up to 0.4 wt% T-Ag-MOF loading (MTA_0.4_), then decline markedly at higher loadings (0.8 wt% and 1.2 wt%) (Supplementary Figure S2). Specifically, the pristine MP_5_ membrane exhibits a tensile strength of 2.26 MPa and an elongation at break of 10.50%, while MTA_0.4_ achieves corresponding values of 2.75 MPa (+21.7% vs. MP_5_) and 26.55% (+153% vs. MP_5_). In contrast, excessive loading detrimentally affects mechanical performance, with the MTA_0.8_ and MTA_1.2_ membranes exhibiting tensile strengths of 1.48 and 0.84 MPa, respectively, together with elongations at break of 19.6% and 8.55%, respectively (Supplementary Figure S2). This enhancement at low loadings was attributed to the BMPN structure at the filler–matrix interface. Within this architecture, Fe^3+^ coordinates with catechol groups and Ag^+^ chelates with phenolate moieties—synergistically promoting uniform nanoparticle dispersion, strengthening interfacial adhesion, and improving stress transfer efficiency across the organic–inorganic interface. Consequently, membrane rigidity and ductility are concurrently enhanced. Conversely, at high loading (1.2 wt%), nanoparticle aggregation disrupts polymer chain continuity, induces interfacial voids, and compromises structural homogeneity—thereby diminishing both the load-bearing capacity and strain tolerance, as evidenced by the marked reductions in both tensile strength and elongation at break.

### 3.4. Antibacterial Properties

**Figure 6:**
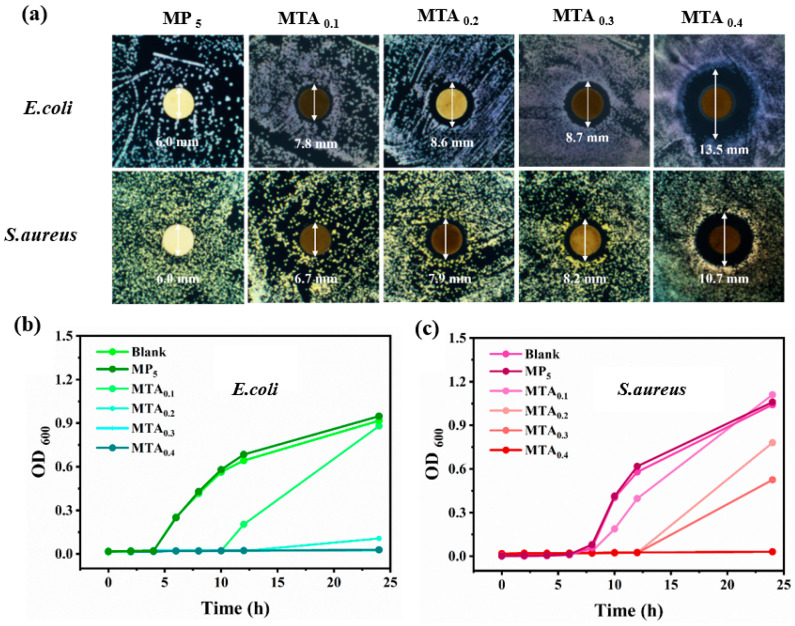
Antibacterial activities of the membranes: (**a**) Representative inhibition zone assays against *E. coli* and *S. aureus* for the pristine PES (MP_5_) and modified membranes with varying T-Ag-MOF loadings; (**b**,**c**) time-dependent bacterial growth kinetics of *E. coli* and *S. aureus* exposed to the different membranes.

The antibacterial efficacy of the T-Ag-MOF/Fe^3+^-modified membrane was subsequently assessed using the disk diffusion method. As depicted in Figure 6a, the MP_5_ membrane exhibits negligible inhibitory effects against both *E. coli* and *S. aureus*. However, the diameter of the inhibition zone increases with the addition of T-Ag-MOF to the membrane. Specifically, the inhibitory zone diameters of MTA_0.1_ against *E. coli* and *S. aureus* are 7.8 and 6.7 mm, respectively. Upon increasing the Ag-MOF dosage to 0.4 wt%, the inhibitory zone diameters expand to 13.5 and 10.7 mm, respectively, demonstrating the excellent antibacterial performance of the MTA_0.4_ membrane. The 24-h growth curves of *E. coli* and *S. aureus* were also examined to evaluate the impact of varying amounts of Ag-MOFs. The results presented in Figure 6b,c further support the aforementioned conclusion, indicating that the optimal antibacterial effect against both *E. coli* and *S. aureus* is achieved for a Ag-MOF dosage of 0.4 wt%.

**Figure 7:**
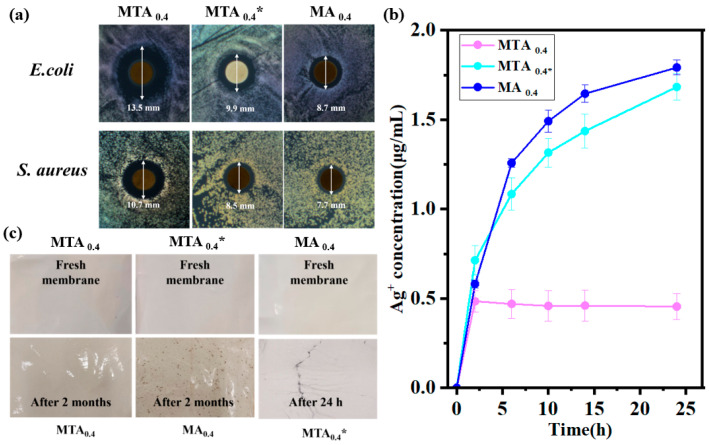
(**a**) Representative inhibition zone images against *E. coli* and *S. aureus* for the MTA_0.4_, MTA_0.4_*, and MA_0.4_ membranes. (**b**) Cumulative Ag^+^ release profiles of the MTA_0.4_, MTA_0.4_*, and MA_0.4_ membranes in ultrapure water. (**c**) Comparative surface morphologies of the MTA_0.4_, MTA_0.4_*, and MA_0.4_ membranes after 2 months of continuous immersion in ultrapure water.

Ag-based antimicrobial materials are known to exhibit broad-spectrum biocidal activity against bacteria, viruses, fungi, and other microorganisms, primarily due to their sustained release of Ag^+^ ions [48,49]. Although Ag^+^ exhibits low intrinsic cytotoxicity and minimal immunogenicity, uncontrolled or burst release may still lead to systemic toxicity and pose potential health risks [36]. To elucidate the mechanism by which BMPN formation modulates both Ag^+^ release behavior and antibacterial efficacy, the structural stability and Ag^+^ leaching profiles of three membrane variants—MTA_0.4_, MA_0.4_, and MTA_0.4_*—were systematically evaluated, each of which was fabricated with a filler loading of 0.4 wt%. The Ag^+^ leaching concentrations were quantified at 2, 6, 10, 14, and 24 h (Figure 7b); MTA_0.4_ exhibits rapid stabilization of Ag^+^ release at ~0.46 μg mL^−1^ (0.48 ± 0.06, 0.47 ± 0.08, 0.46 ± 0.09, 0.46 ± 0.09, and 0.46 ± 0.07 μg mL^−1^, respectively), whereas MA_0.4_ shows a continuous increase from 0.72 ± 0.03 to 1.59 ± 0.03 μg mL^−1^ over the same period. In contrast, MTA_0.4_* displays intermediate but non-stabilized release kinetics (0.61 ± 0.04, 0.79 ± 0.05, 0.92 ± 0.03, 1.03 ± 0.04, and 1.18 ± 0.06 μg mL^−1^). Consistent with these release patterns, MTA_0.4_ generates significantly larger inhibition zones against *E. coli* and *S. aureus* than either MA_0.4_ or MTA_0.4_*, as illustrated in Figure 7a. These results collectively demonstrate that BMPN integration—achieved via TA–Fe^3+^ coordination—enables precise temporal control over Ag^+^ delivery. Specifically, it suppresses initial burst release, maintains a stable low-level flux, enhances antibacterial efficacy, and concurrently mitigates risks associated with excessive ion leaching and secondary contamination.

The influence of BMPN on the structural stability of the T-Ag-MOF/Fe^3+^/PES composite membranes is illustrated in Figure 7c. All three freshly fabricated membranes exhibit uniform, defect-free surfaces with high morphological integrity. Notably, the MTA_0.4_* membrane—fabricated using T-Ag-MOF in the absence of Fe^3+^—shows extensive deposition of gray particulates on its surface after only 24 h of immersion in ultrapure water, providing direct morphological evidence of severe T-Ag-MOF leaching. In contrast, the MA_0.4_ membrane—prepared using the unmodified Ag-MOF (i.e., lacking TA functionalization) and Fe^3+^ in the coagulation bath—develops conspicuous brown aggregates after two months of continuous water exposure, revealing progressive detachment of the MOF from the PES matrix under sustained hydration. Meanwhile, the MTA_0.4_ membrane—synthesized in the presence of both T-Ag-MOF and Fe^3+^—maintains its original surface smoothness, microstructural homogeneity, and dimensional integrity throughout the entire two-month immersion period; no filler migration, aggregation, delamination, or surface deterioration was observed.

These results collectively demonstrate that the BMPN—formed in situ via synergistic coordination of TA with both Ag^+^ (on the MOF surface) and Fe^3+^ (diffusing into the nascent polymer matrix)—significantly enhances interfacial compatibility between the hydrophilic T-Ag-MOF and inherently hydrophobic PES matrix, thereby conferring exceptional aqueous stability to the composite membrane. Mechanistically, TA acts as a multifunctional interfacial mediator: (i) it first anchors onto the T-Ag-MOF surface through strong coordination with surface-bound Ag^+^ species, forming a conformal metal–polyphenol layer; and (ii) during phase inversion, it rapidly chelates Fe^3+^ ions infiltrating the polymer matrix, leading to the formation of a robust, cross-linked BMPN both at the membrane surface and within the porous substructure. This dual-site coordination reinforces filler–matrix adhesion and effectively suppresses interfacial delamination.

The inferior stability observed in the control membranes stems from fundamental interfacial incompatibilities. Specifically, the unmodified Ag-MOF in the MA_0.4_ membrane lacks phenolic anchoring groups, resulting in weak physical adsorption onto the PES matrix, poor dispersion, and susceptibility to detachment under hydraulic shear and osmotic stress. Conversely, although the MTA_0.4_* membrane incorporates the TA-modified Ag-MOF, the absence of Fe^3+^ in the coagulation bath precludes trivalent metal-mediated crosslinking, thereby preventing the formation of a mechanically robust BMPN. Consequently, the resulting architecture lacks sufficient cohesive strength to withstand prolonged hydration-induced swelling and hydrodynamic forces. Collectively, these findings indicate that TA surface modification alone is insufficient for ensuring long-term membrane stability. Rather, the in situ, spatiotemporally controlled formation of a bimetallic (Ag/Fe) polyphenol network—enabled by the concurrent coordination of TA to Ag sites on the MOF and Fe^3+^ ions within the PES matrix—is essential for achieving covalent-like interfacial integration, synergistic structural reinforcement, and sustained ion-release functionality. As illustrated in Figure 7b, TA functions not as a passive “intermediate bridge,” but as an active, dual-site “coordination bridge” that simultaneously chelates Ag^+^ centers on the MOF surface and coordinates Fe^3+^ ions within the polymer matrix, thereby resolving the intrinsic incompatibility between the inorganic filler and organic polymer phases.

As shown in Figure 7c, visible mottling is evident on the surface of the MA_0.4_ membrane after 2 months, which indicates the leakage of Ag-MOFs structures. Although Ag^+^ exhibits low cytotoxicity and elicits only a minimal immune response, the sudden release of large quantities of this metal ion poses a risk of toxicity [48,49]. The pretreatment of Ag-MOFs with TA not only alters the surface hydrophobicity but also protects the metal ions, effectively delaying their release from the MOF structure [36]. However, immersion of the MTA_0.4_* membrane (without TA–Fe^3+^ chelation) in deionized water for 24 h results in an overflow of Ag-MOFs particles from the membrane (Figure 7c). These findings suggest that the construction of BMPNs enables Ag^+^ release control superior to that achieved through the simple chelation of TA with Ag-MOFs. As detailed above, TA acts as an “intermediate bridge” in the formation of BMPNs, combining with the Ag-MOF and coordinating with Fe^3+^ during the phase transformation process. As phase transformation occurs, the BMPN structure anchors T-Ag-MOFs both on the membrane surface and inside the membrane, resolving the incompatibility between inorganic particles and organic membranes, and ultimately enhancing the stability of the modified membranes.

## 4. Conclusions

In this study, hydrophilic antifouling PES membranes incorporating BMPNs were fabricated. These BMPNs consisted of hydrophilic T-Ag-MOFs particles integrated with TA–Fe^3+^ coordination layers, which were formed via synergistic chelation and the mild acidity of TA. The effects of the T-Ag-MOF loading on the resulting membrane morphology and physicochemical properties were systematically investigated. At an optimal loading of 0.4 wt%, the resulting T-Ag-MOF/Fe^3+^/PES composite membrane exhibited a pure water permeability of 593.4 L m^−2^ h^−1^ bar^−1^—1.77-fold higher than that of the pristine PES membrane—while maintaining a high BSA rejection capability of 96.5%. Critically, the strategic integration of TA as a bifunctional interfacial bridge (coordinating simultaneously with Ag^+^ on the T-Ag-MOF and Fe^3+^ within the PES matrix) enabled the in situ formation of a robust, cross-linked BMPN architecture. This structure markedly enhanced interfacial compatibility between the hydrophilic T-Ag-MOF filler and inherently hydrophobic PES polymer, thereby establishing covalent-like filler–matrix adhesion. Consequently, the composite membrane delivered synergistic multifunctionality, including exceptional long-term aqueous stability, precisely tuned Ag^+^ release kinetics (effectively suppressing burst release while sustaining a therapeutically relevant, low-level flux), and potent antibacterial activity—without compromising biosafety—as evidenced by negligible uncontrolled ion leaching and a lack of structural degradation under prolonged hydration. Collectively, the abundant phenolic hydroxyl groups in TA serve not only as versatile molecular bridges but also as active coordination centers that drive the assembly of robust BMPNs with Fe^3+^ and Ag^+^. This coordination-based design significantly enhances interfacial adhesion and compatibility between inorganic fillers and PES membranes, thereby providing a novel approach for the fabrication of multifunctional PES-based membranes.

## Figures and Tables

**Table 1:** Compositions of the casting solutions and coagulation baths used to prepare the BMPN membranes.

Membrane	Casting Solution (wt%)					Coagulation Bath (mg mL^−1^)
	T-Ag-MOF	Ag-MOF	PES	NMP	PVP	Fe^3+^
MP_5_	0	0	17.0	78.0	5.0	1.0
MTA_0.1_	0.1	0	17.0	78.0	5.0	1.0
MTA_0.2_	0.2	0	17.0	78.0	5.0	1.0
MTA_0.3_	0.3	0	17.0	78.0	5.0	1.0
MTA_0.4_	0.4	0	17.0	78.0	5.0	1.0
MTA_0.4_*	0.4	0	17.0	78.0	5.0	0
MA_0.4_	0	0.4	17.0	78.0	5.0	1.0

**Table 2:** Fouling resistance performance of the pristine PES and T-Ag-MOFs/Fe^3+^–modified membranes.

Membrane	FRR (%)	R_r_ (%)	R_ir_ (%)	R_t_ (%)
MP5	57.50 ± 2.50	35.16 ± 1.41	42.50 ± 2.50	77.66 ± 1.09
MTA0.1	66.31 ± 0.35	43.17 ± 1.51	33.69 ± 0.35	76.86 ± 1.86
MTA0.2	67.65 ± 2.35	43.52 ± 1.48	32.35 ± 2.35	75.87 ± 0.87
MTA0.3	75.24 ± 0.69	50.49 ± 3.22	24.76 ± 0.69	75.25 ± 2.53
MTA0.4	78.68 ± 0.35	53.31 ± 1.69	21.32 ± 0.35	74.62 ± 2.04

## Data Availability

The data supporting the findings of this study are available from the corresponding author upon request.

## Supplementary Materials

The following supporting information can be downloaded at: https://www.mdpi.com/article/10.3390/polym18111326/s1, Figure S1: SEM image of (a,b) Ag-MOF, and (c,d) T-Ag-MOF; Figure S2: Tensile stress-strain curves of MP_5_ and different T-Ag-MOF/Fe^3+^/PES composite membranes.
