# Supplementary material for: Enhancing the Water Flux and Antifouling Properties of PES Membranes via the Construction of a Bimetallic Polyphenol Network
Source: Polymers (Basel). 2026 May 27;18(11):1326. doi: 10.3390/polym18111326 (PMC13259196; doi:10.3390/polym18111326)
Supplement: Supplementary file 1 [file polymers-18-01326-s001.zip › polymers-4301162-supplementary.pdf]

Supporting Information For

Enhancing the water flux and antifouling properties of PES membranes via the construction of a bimetallic polyphenol network

Yubin Lin [1], Xiaoxue Xiao [2], Wenqiang Deng [1,2], Wei Mao [1,2], Cui Wei [1,2,*] and Jinghong Zhou [1,2,*]

1 Light Industry and Food Engineering College, Guangxi University, Nanning 530004, PR China

2 Guangxi Key Laboratory of Clean Pulp & Papermaking and Pollution Control, Nanning 530004, PR China

* Correspondence: xiaoxiaoxue@boe.com.cn (Xiaoxue Xiao); 2316301046@st.gxu.edu.cn (Cui Wei); jhzhoudou@gxu.edu.cn (Jinghong Zhou)

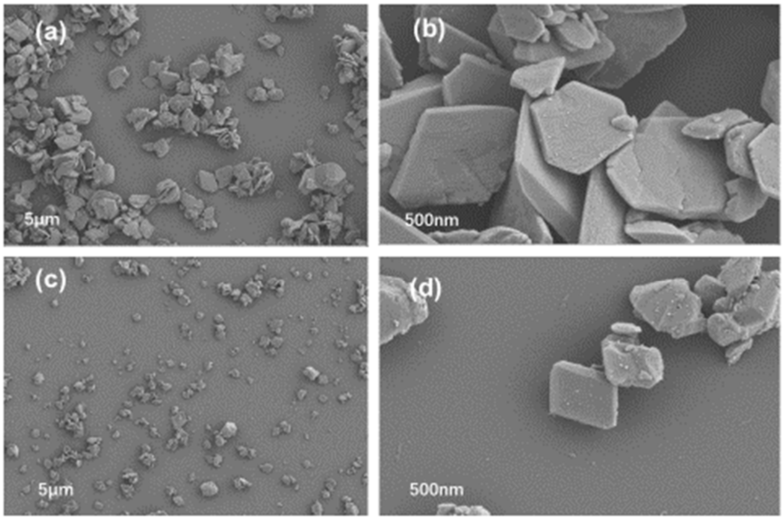

Figure S1. SEM image of (a, b) Ag-MOF, and (c, d) T-Ag-MOF.

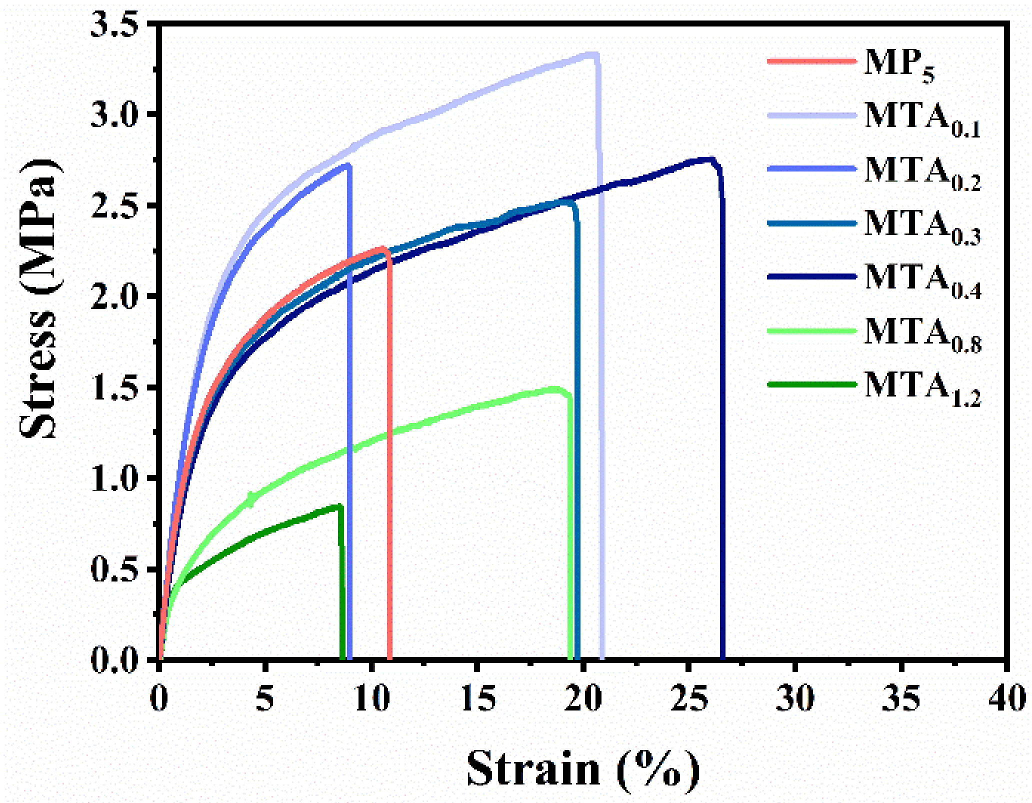

Figure S2. Tensile stress-strain curves of MP_5 and different T-Ag-MOF/Fe^{3+}/PES composite membranes
